# The Relationship Between Chinese EFL Learners’ Resilience and Academic Motivation

**DOI:** 10.3389/fpsyg.2022.871554

**Published:** 2022-05-06

**Authors:** Bo Zhang

**Affiliations:** College of Foreign Languages, Zhoukou Normal University, Zhoukou, China

**Keywords:** positive psychology, Chinese EFL learners, constant motivation, academic motivation, resilience

## Abstract

With the recent adoption of Positive Psychology in foreign language education, academic motivation and resilience as secondary components of positive psychology have started to receive academic attention. Undoubtedly, students require constant motivation because learning a foreign language is a long-term effort especially in the first stage that they usually lose their interest and motivation. When students are academically motivated, they can put high effort to learn the language. This study aims to inspect the relationship between Chinese EFL learners’ resilience and academic motivation. To this end, 482 students from different colleges and universities in China were selected and they completed the resilience and academic motivation questionnaires. Spearman’s rho index and multiple regressions were run for data analyses. Results of the study indicated that there is a positive and significant association showing a relationship between these two constructs. Moreover, two components of motivation, namely interjected regulation and external regulation by attendance proved to be the best predictors of learners’ resilience. The paper concludes with some pedagogical implications; for instance, motivation can be considered as a primary point for the progress of resilience for the next steps of language success.

## Introduction

Language education has a long way to go; therefore, students must have sufficient motivation to attain the goal. The degree of motivation of a language student can alter during this journey because of numerous disturbances that influence his or her motivation (Dörnyei and Ryan, 2015). In recent years, the constructive function of motivation has been debated in past research (Dörnyei and Ushioda, 2013) as one of the singular contrasts that affect the effectiveness of second language education. Fundamentally, the concept of motivation has been the center of human achievement and accomplishment for a long time, not only in personal life but also in the scholastic one (Gopalan et al., 2018). Motivation has been extensively investigated in the education milieu from a variety of aspects and viewpoints, confirming with one accord the influence of this concept on students’ success and education (Al-Hoorie, 2017). Motivation is the primary motive behind people selecting an action, the duration of proceeding with that action, and the degree of resolution in seeking that action (Dörnyei and Skehan, 2003). Nonetheless, in the setting of EFL, where English is taught as a compulsory course in schools, students are prone to lose interest in studying English and frequently encounter demotivation (Kikuchi, 2013). Motivation by itself is insufficient for reaching the final goal of learning since language students encounter demotivating issues like the powerful essence of learning a language, especially EFL, and factors about teachers in the lengthy process of language education (Kikuchi, 2015).

Moreover, there is widespread consensus that the impetus for learners’ scholastic educational manner is normally motivation and while motivation is essential in the foreign language learning process, resilience in face of problems or difficulties and the ability to use many problem-solving tactics are also vital to the success of foreign language students (Ushioda, 2008). In this respect, persisting in learning the language is vital for every student to reach a proper level of proficiency, no matter what challenges exist. Indeed, among other factors, students’ success can be attributed to bounce back, the ability to withstand difficulty, and recuperating from stress, which can be referred to as resilience (Nguyen et al., 2015). It means the totality of a person’s ability to overcome problems and make efforts in difficult times (Kim and Kim, 2017). The function of resilience in the educational domain has been explained as a way to stop school dropout despite the challenges learners encounter, like being poor, experiencing brutality, and having family-related anxiety (Condly, 2006). Scholastic resilience can be characterized as learners’ capability of successfully managing failures, difficulties, hardships, and tension in the scholastic environment (Martin and Marsh, 2006). Furthermore, as a significant factor in PP, resilience alludes to the pliability of those who deal with different troubles and difficulties of life and recover from hardships and misery (Dent and Cameron, 2003). Resilience has been shown to have a significant function in lessening the anxiety and worry that comes along with studying a foreign language, as a result, improving the language education experience (Chaffee et al., 2014). Resilience can have a significant function in language education given its constructive function in conquering life difficulties.

According to the previous studies, it can be presumed that students with a high level of motivation in language education will put more endeavor and conquering demotivating elements when they are completely provided with resilience. Lately, there has been increasing interest in the application of positive psychology (PP) in the domains of language learning and instruction, and the motivation and resilience of language education have started to attract scholastic attention as sub-elements of PP (Oxford, 2016; Dewaele et al., 2019; MacIntyre et al., 2019; Wang et al., 2021). Nonetheless, few experiential studies have attended to what results in the advancement of strong points and qualities of language students, like resilience, joy, and positivity inside a constructive mental frame (MacIntyre and Mercer, 2014; Oxford, 2016; Wang and Guan, 2020).

Grounded on the literature review, and due to the importance of motivation and the resilience of learners in language education, it appears to be noteworthy to pay attention to these two concepts to provide significant advantages in this area. Moreover, in language learning, the connection between learner’s resilience and language education motivation has been poorly studied. Therefore, based on the remarkable role of motivation in learning, on the one hand, and learners’ resilience, on the other hand, it seems necessary to search the relationship between these two concepts in language learning. Several inquiries have examined the motivation-academic resilience relationship, which has merely and generally carried out correlation or stepwise regression in terms of education and not in terms of teaching language (Martin, 2002; Kim and Kim, 2017; Shin and Kim, 2017; Wang et al., 2022). Indeed, the correlation between learners’ resilience and academic motivation has not been scrutinized in the realm of language teaching so far. Likewise, the results can tell us about the function and aspects of learners’ motivation in language learning; thus, both educators and students may benefit from the outcomes. Furthermore, the results add knowledge regarding learners’ resilience and help learners maintain their motivation. Accordingly, the present research intended to fill in this gap by investigating the relations between students’ resilience and language learning motivation in the Chinese EFL setting. Accordingly, the succeeding research questions are articulated:

Q1. Is there any significant relationship between Chinese EFL learners’ motivation and resilience?
Q2. Which component(s) of Learners’ academic motivation can best predict their resilience?

## Review of the Literature

### Resilience

A concept about psychology and human development theory is resilience defined as a dynamic process that involves positive adaptation in the intense adversity situation or the ability of people to resist in the face of adversity and adapt to their setting (Luthar et al., 2000; Connor and Davidson, 2003; Xue, 2021; Wang et al., 2022). Because of its descriptive role, one can consider resilience an important personal differentiator when it is necessary to explain through study why some outperform others in the management of challenges (White et al., 2010). Resilience is defined as a progression in preference to a trait: a dynamic, transferred process in people (internal) and between people and their setting (external) for sources and supports to identify and adapt themselves as healthy during difficulties, trauma, threats, and/or daily distress (Truebridge, 2014). In a study by Wagnild and Collins (2009), numerous kinds of resilience and measures were built. Personal resilience refers to ones’ capability to recuperate from problems, to change easily, and to get soft, instead of fighting, when encountering hardship. Personal resilience means a multifaceted construct encompassing certainty and capability for acceptance, adaptation, and recovering from difficulties. There are some traits of personal resilience, namely, self-confidence, adaptation, risk-taking, solving problems, robustness, self-regulation, social skills, self-direction, optimism, cooperation, curiosity, tending to self-regulate, perceiving and appreciating growth resulting from difficult situations, beliefs, hopes, contemplation, creativity, initiative, and an amazing sense of fun backing a realistic outlook on living.

### Motivation

Motivation is considered as a determinant influencing what learners conduct and how well they fulfill it. A person can be inspired by both external and internal motivation, where the second one leads to action or performance of certain behaviors (Utvaer and Haugan, 2016). Academic motivation refers to motivation that starts and directs learners to be more fascinated in the process of learning (Lavasani et al., 2011; Wang and Guan, 2020). Ushioda (2008) preserved that Motivation deals with the rationale of people who make specific choices, engage in activities, and persist in seeking them. Motivation controls the degree of intensive personal involvement in second language education. To describe the function of motivation in people, Deci and Ryan (2000) proposed (SDT) where the human behavior motivation is categorized according to the extent of internalization. According to this theory, people manage their behavior and personality growth when experiencing various “types of motivation” (Ryan and Deci, 2020). In other words, SDT tries to describe motivation from a personal point of view which ignores external interference, while target theory pays attention to goals or targets determined by people. The two theories offer individualist approaches that emphasize intrinsic motivation. Learners learning English in an educational setting can experience these types of motivation when searching for actual foreign language learning tactics (Lee and Bong, 2019). The societal intellectual frameworks regard motivation as a complex occurrence. Learners can be motivated through different means, and rating tools that give a singular universal “motivation” outcome for learners can be deceptive (Linnebrink and Pintrich, 2002). The theory of self-determination specifically emphasizes the difference between inherent motivation, interventional control, specified control, outer control, and motivation (Ratelle et al., 2007).

## Method

### Participants

A sample of 482 EFL learners was collected *via* Wechat employing Wenjuanxing from different colleges and universities with the majority in Henan Province (479/99.1%) and other provinces (Fujian, Jiangsu, Heilongjiang; 3/0.9%) in China. To generalize the research results, both genders (females = 428; males = 54) were surveyed with their age ranging from 16 to 40, including 106 junior college EFL learners (21.9%), 373 bachelor EFL learners (77.3%), one master EFL learners (0.2%) and two doctoral EFL learners (0.6%) respectively. Informed consent was given to all the participants. All of the data we collected was based on the respondents’ willingness.

### Instruments

#### Motivation Questionnaire

The questionnaire used in this study was the 28-item motivation questionnaire (Vallerand et al., 1989). The five-factor variant of the research survey was utilized since it is consistent with the initial suggestion by Deci and Ryan (1985). The survey was examined for internal consistency. The surveys, as well as the written informed consent documents, were given out to students in medical school by the scholar himself. The objective of the research was directly proposed and enough time (30 min) was allocated to every participant to finish the survey. Its components are amotivation, introjected regulation, external regulation by attendance, external regulation by social interaction, identified regulation, integrated regulation, and intrinsic motivation. The internal consistency of the questionnaire was 0.883 calculated by Cronbach’s Alpha.

#### Resilience Scale

The resilience scale developed by Lereya et al. (2016) is a 40-item measure containing 12 subscales assessing learners’ insights of their distinct features along with protecting aspects rooted in the milieu. The frequency of each item was rated on a 5-point scale (From 1—never, 2—seldom, 3—sometimes, 4—often, 5—always). It is worth mentioning that the internal of the questionnaire was calculated by Cronbach’s Alpha and it was 0.971.

### Data Collection Procedures

To meet the purposes of this study, by distributing questionnaires online *via* Wenjuanxing (an online questionnaire program to collect the data) *via* Wechat, data was collected smoothly in the middle of December. Altogether, 482 valid questionnaires were gleaned from different provinces in China and one foreign country. To upsurge the reliability and validity of the sample, all participants were fully notified of how to fill out the questionnaires and how to give validated answers and assured that their responses and personal information were only used to meet the objectives of the research would remain confidential. As the participants made no contact with the research, there was no conflict of interest between the research and correspondents. Then, the collected responses were double-checked for potential limitations before being processed by SPSS software for further statistical analysis, which was, at the final step, conducted for the exploration of answers to research questions.

### Data Analysis

To answer the first research question of the present research on the relationships between the two key terms of the study, the Spearman’s rho index was employed while for the second research question, multiple regression is used. The data were analyzed through the Statistical Package for Social Science (SPSS), version 20.0.

## Results

The purpose of this research is to inspect the role of Chinese EFL learners’ motivation and teachers’ resilience. Consistent with the research questions of the current study, Pearson’s Product-moment correlation coefficient and linear regressions were done.

Q1. Is there any significant relationship between Chinese EFL learners’ motivation and resilience?

Table 1 shows the reliability of Students’ resilience and their motivation questionnaires. The results revealed that the questionnaires enjoy highly satisfactory reliability indices (0.88, 0.97).

**TABLE 1:** Reliability of the questionnaires.

Cronbach’s Alpha	*N* of items
0.883	28
0.971	40

The normality test assumes that the data are normal. The Kolmogorov-Smirnov index for learners’ motivation showed that the data are normal (sig. = 0.200) since no significant value was detected. On the other hand, Table 2 shows that the data for learners’ resilience questionnaire are not normal since sig. = 0.005 < 0.05. Consequently, the data analysis procedure for running correlation should be the Spearman rho index.

**TABLE 2:** Normality Test for questionnaires.

	Kolmogorov-Smirnov^a^			Shapiro–Wilk		
		
	Statistic	df	Sig.	Statistic	df	Sig.
Motivation	0.032	482	0.200*	0.994	482	0.044
Resilience	0.051	482	0.005	0.977	482	0.000

*This is a lower bound of the true significance. ^a^Lilliefors significance correction.

Table 3 displays the correlations between learners’ motivation and their resilience. Considering the negative index (−0.153) for this relationship, it can be concluded that if learners’ motivation increases, their resilience will decrease. Moreover, this relationship is significant since the *p*-value is 0.001 < 0.05.

**TABLE 3:** Correlations for learners’ motivation and their resilience.

			Motivation	Resilience
Spearman’s rho	Motivation	Correlation coefficient	1.000	−0.153**
		Sig. (2-tailed)	.	0.001
		*N*	482	482
	Resilience	Correlation coefficient	−0.153**	1.000
		Sig. (2-tailed)	0.001	.
		*N*	482	482

**Correlation is significant at the 0.01 level (2-tailed).

Q2: Which component(s) of Learners’ academic motivation can best predict their resilience?

To identify which of the subscales of learners’ academic motivation can best predict their resilience, the researcher ran a multiple regression. The output tables are as the followings:

For ease of understanding, the following abbreviations were provided in Table 4.

**TABLE 4:** Correlations among the components of motivation and resilience.

		R	AM	IR	ERBA	ERBS	IdR	InR	IM
Pearson Correlation	R	1.00	−0.08	−0.18	−0.15	0.04	0.06	0.02	−0.01
	AM	−0.08	1.00	0.60	0.55	0.37	0.46	0.46	0.42
	IR	−0.18	0.60	1.00	0.66	0.28	0.41	0.47	0.51
	ERBA	−0.15	0.55	0.66	1.00	0.44	0.49	0.46	0.41
	ERBS	0.04	0.37	0.28	0.44	1.00	0.61	0.47	0.28
	IdR	0.06	0.46	0.41	0.49	0.61	1.00	0.60	0.37
	InR	0.02	0.46	0.47	0.46	0.47	0.60	1.00	0.48
	IM	−0.01	0.42	0.51	0.41	0.28	0.37	0.48	1.00
Sig. (1-tailed)	R		0.04	0.00	0.00	0.21	0.08	0.29	0.42
	AM	0.04		0.00	0.00	0.00	0.00	0.00	0.00
	IR	0.00	0.00		0.00	0.00	0.00	0.00	0.00
	ERBA	0.00	0.00	0.00		0.00	0.00	0.00	0.00
	ERBS	0.21	0.00	0.00	0.00		0.00	0.00	0.00
	IdR	0.08	0.00	0.00	0.00	0.00		0.00	0.00
	InR	0.29	0.00	0.00	0.00	0.00	0.00		0.00
	IM	0.42	0.00	0.00	0.00	0.00	0.00	0.00	
*N*		482	482	482	482	482	482	482	482

R = Resilience, AM = amotivation, IR = Interjected Regulation, ERBA = external motivation by attendance, ERBS = external motivation by social, IdR = identified regulation, InR = integrated regulation, IM = intrinsic motivation.

Table 4 shows the correlations among the components of motivation and resilience. The Pearson correlations, regardless of the direction of the relationship, show that interjected regulation and external regulation by attendance have the highest indices (0.18, 0.15). Consequently, the relationship between these components and learners’ resilience is significant (0.000).

Table 5 shows whether or not this model is appropriate for learners’ motivation and resilience. R square index shows that learners’ academic motivation can predict only seven percent of their resilience.

**TABLE 5:** Model summary for the components of motivation and resilience.

Model	R	R Square	Adjusted R Square	Std. Error of the Estimate	Durbin-Watson
1^b^	0.270^a^	0.073	0.05	28.84	1.89

*^a^Predictors: (Constant), AM, IR, ERBA, ERBS, IdR, InR, IM. ^b^Dependent variable: resilience.*

As indicated in Table 6, a significant regression equation was found [*F*(7, 474) = 5.32, *p* = 0.000] with an *R*^2^ of 0.073.

**TABLE 6:** ANOVA test for the components of motivation and resilience.

Model		Sum of squares	df	Mean square	*F*	Sig.
1^a^	Regression	31,011.55	7	4,430.22	5.32	0.000^b^
	Residual	394,483.84	474	832.24		
	Total	425,495.39	481			

*^a^Dependent variable: resilience. ^b^Predictors: (Constant), AM, IR, ERBA, ERBS, IdR, InR, IM.*

Table 7 shows the best predictor(s) of learners’ academic motivation, which includes seven components, for learners’ resilience. To identify the best predictors standardized Beta coefficients were checked. Interjected regulation (*B* = 0.20, *P* = 0.000), and external regulation by attendance (*B* = 0.15, *P* = 0.01) proved to be the best predictors of learners’ resilience.

**TABLE 7:** Coefficients for the components of motivation and resilience.

		Unstandardized coefficients		Standardized coefficients		
			
Model		B	Std. Error	Beta	*t*	Sig.
1^a^	(Constant)	140.46	7.73		18.15	0.000
	AM	–0.02	0.32	–0.00	–0.07	0.93
	IR	–1.00	0.32	–0.20	–3.04	0.00
	ERBA	–1.40	0.57	–0.15	–2.45	0.01
	ERBS	0.300	0.68	0.02	0.43	0.66
	IdR	1.348	0.57	0.15	2.34	0.02
	InR	0.58	0.58	0.06	0.99	0.32
	IM	0.78	0.60	0.07	1.29	0.19

*^a^Dependent variable: resilience.*

## Discussion

The purpose of the study is to explore the relationship between Chinese EFL learners’ resilience and academic motivation. The results of the study proved their significance relationship between these two variables. Academic resilience is a factor that improves the success of the school, as well as academic competence and school experience. That is, resilient learners show high academic performance and motivation regarding achievement without the loss of a positive attitude toward learning and school despite a tedious school setting, namely some risk factors such as declined performance and dropout (Cassidy, 2015). According to the above-mentioned results, resilience is highly significant in high educational motivation and even greater rates of achievement. Therefore, resilient people can cope with many hard circumstances since they need to be capable of coping with and adjusting to fluctuations that take place. A person’s condition when he or she encounters challenges cannot be evaded; however, individuals who possess resilience are capable of conquering different issues using their own tactics and methods. This study investigated the association between dimensions of students’ motivation and their resilience among Chinese EFL learners. The findings of the study specified that each aspect of learners’ motivation had a noteworthy association with their resilience while the interjected regulation and external regulation were significant predictors of their resilience. In interjected regulation, even though people have inner motives, outer elements are the origin of their manners through the declaration of people’s self-perception or the devising to create stress and self-reproach. The outcomes of the study are in line with those of Shin and Kim (2017) that indicated the function of resilience on intrinsic motivation and the ideal language of Korean learners. The results also support that of Chang (2004) who determined that students with high resilience enjoyed higher interest and satisfaction with learning, which shows that resilience and intrinsic motivation are positively correlated. Additionally, the findings are congruent with those of Martin and Marsh (2006) study which proposed that academic resilience positively affects pleasure with school era and participation in class. The study carried out by Rouse (2001) specified that students with high resilience are less demotivated when learning English, which is consistent with previous research that the higher the resilience, the more sustained learning motivation. It can be supposed that learners with high motivation to learn can exert more effort and overcome the factors that decrease motivation when they have sufficient resilience (Kim and Kim, 2017).

## Conclusion and Implications

This research can provide an understanding of the evolution of language instruction and education, specifically in terms of comprehending resilience and how it is linked to learners’ motivation. Indeed, learners’ academic resilience in the learning procedure can be improved by enhancing the learners’ internal and external motivation. They can also develop it through initiatives to understand the needs and goals of learning, among other preparations that complete the strengthening of other academic resilience. Resilience has a positive effect on success in various areas, including motivated behavior. Learners’ resilience is triggered to fit particular areas that require knowledge and skills when facing challenges of language learning. Enhancing resilience will help to improve the motivating behavior intensity and language competency due to the effect of resilience on motivated behavior (Oxford, 2011). When students witness the benefits of language learning and relate it to their own life, they get higher satisfaction with the process of language learning, leading to an increase in students’ level of resilience and motivated behavior. It will be also helpful to have enough awareness about the issue and allow students to embrace the inevitable challenges they will face when learning a language (Oxford, 2011). Moreover, students’ determination, positive attitude, and motivation can aid them to overcome difficulties and actively seek external resources they can use to overcome them. Regarding the findings of the present research, educators, teacher educators, and policymakers of language should explore the importance of ways to increase educators’ motivation to increase the resilience and persistence of English educators. It is hoped that this research will be useful for educators who try to produce motivation by using the strengths of learners as a beginning point for learning. Through educators’ responsibility in active engagement, they can increase their learners’ motivation for gaining higher academic outcomes. Resilience aid learners in successfully coping with academic problems, stress, and learning pressures in the process of learning, and through which, some insight are presented for teachers who are looking for situations to create an optimal learning environment for all their learners.

Another pedagogical use of the present research relates to the type of content and methods used by educators to enhance the motivation of language learners. The use of interesting, persuasive, informative, and less cognitively challenging material can enhance the motivation of students. Educators and syllabus designers may better understand the development procedures and assisting factors needed for shaping and nurturing student resilience during language learning. Furthermore, a teaching method by which educators inspire language students to actively engage in classroom activities and providing opportunities to employ language can give students a sense of accomplishment, which instead can enhance their motivation. A suggestion made out of the upshots of the present study is that motivation can be regarded as a beginning point and primary stage for the development of resilience for the next steps of language achievement.

Based on these results, educators and school administrators are encouraged to develop specific programs to teach learners resilience skills. In addition, it is recommended that other studies in the same field be conducted together to investigate intrinsic factors predicting academic motivation besides academic resilience. Considering the results of this study, counseling services at universities and schools should prioritize research into factors that straightly affect learners’ learning outcomes, namely, academic resilience and increased learning motivation. Similarly, one should prioritize research that motivates learners and strengthen internal resources, and also future qualitative studies should be done to inspect students’ resilience through interviews to detect the variability of their resilience over time. Besides investigating the relationship between resilience and motivation in other learning contexts, further research could inspect the association between students’ resilience and other emotional concepts such as anxiety, stress, apprehension, particularly in the Chinese situation.

## Ethics Statement

The studies involving human participants were reviewed and approved by Zhoukou Normal University Academic Ethics Committee. The patients/participants provided their written informed consent to participate in this study.

## Author Contributions

The author confirms being the sole contributor of this work and has approved it for publication.

## Conflict of Interest

The author declares that the research was conducted in the absence of any commercial or financial relationships that could be construed as a potential conflict of interest.

## Publisher’s Note

All claims expressed in this article are solely those of the authors and do not necessarily represent those of their affiliated organizations, or those of the publisher, the editors and the reviewers. Any product that may be evaluated in this article, or claim that may be made by its manufacturer, is not guaranteed or endorsed by the publisher.
